# No relationship between the digit ratios (2D:4D) and salivary testosterone change: Study on men under an acute exercise

**DOI:** 10.1038/s41598-020-66915-9

**Published:** 2020-06-22

**Authors:** Marta Kowal, Piotr Sorokowski, Agnieszka Żelaźniewicz, Judyta Nowak, Sylwester Orzechowski, Grzegorz Żurek, Alina Żurek, Anna Juszkiewicz, Lidia Wojtycka, Wiktoria Sieniuć, Małgorzata Poniatowska, Karolina Tarnowska, Kaja Kowalska, Katarzyna Drabik, Patrycja Łukaszek, Krzysztof Krawczyk, Tadeusz Stefaniak, Natalia Danek

**Affiliations:** 1 Institute of Psychology, University of Wrocław, Wrocław, Poland; 2 Department of Human Biology, University of Wrocław, Wrocław, Poland; 3 University School of Physical Education in Wrocław, Wrocław, Poland; 4 Maria Curie-Skłodowska University, Lublin, Poland

**Keywords:** Anthropology, Evolutionary theory

## Abstract

The digit ratio (2D:4D) is said to be a potential marker of exposure to prenatal sex steroids. Some studies suggest that the 2D:4D is also linked with the testosterone response to challenging situations due to organizational effect of prenatal hormonal milieu on adult endocrine functioning. However, up to date, there were only four studies (conducted on small samples) that examined the 2D:4D and the testosterone response to a challenging situation (i.e. physical exertion or aggressive context). Here, we examined the relationship between the 2D:4D and the testosterone change under an acute exercise among 97 men. We found that the digit ratios (the right 2D:4D, the left 2D:4D, and the right minus left 2D:4D) were neither predictors of pre-exercise testosterone, nor the change in testosterone level after a cycling task. Our results add a contradictory to previous studies evidence in a discussion on the links of the 2D:4D and the testosterone change.

## Introduction

More than 100 years ago, a difference in the length of the 2^nd^, index finger (2D) and the 4^th^, ring finger (4D) has been described^1^. Further studies provided evidence that men and women vary when it comes to the magnitude of this difference^2^. The ratio between the length of the 2^nd^ and the 4^th^ finger (2D:4D) has been reported to be smaller among men compared to women (men have longer the 4^th^ finger than the 2^nd^ finger) (but see also:^3,4^). Since then, researchers have been interested in the origins and implications of the 2D:4D sexual dimorphism.

Manning and colleagues^5^ suggested that the difference between the 2D:4D among men and women develops during gestation under prenatal sex steroids control. The 2D:4D is said to be directly connected with the exposure to androgens in the uterus (with the lower digit ratios associated with the exposure to higher levels of testosterone, and higher digit ratios associated with the exposure to lower levels of testosterone). Hence, the 2D:4D is perceived as an index of prenatal testosterone level. However, correlational studies on the relationship between the 2D:4D and the prenatal testosterone level conducted on human fetuses brought conflicting results^6–11^. Similar, mixed results have been found in experimental studies on animals^12,13^, thus, it is not surprising that such links are perceived as questionable and unclear^14–16^. Even more speculative is that some researchers presume that the low 2D:4D may reflect higher adult testosterone^17–27^. Interestingly, only a few studies reported a negative link between the 2D:4D and adult sex hormone levels^5,18–21,23^, whereas a meta-analysis conducted by Hönekopp *et al*.^28^ and Zhang *et al*.^15^ found no such association.

Because there are many mixed findings on the mechanism of the observed differences in digit ratios^12–14,28^, and at the same time, there is a constantly growing body of literature showing positive associations between the 2D:4D and, for instance, psychological or physiological^29,30^ characteristics, new hypotheses explaining the potential relationship between the 2D:4D and aforementioned traits are being suggested.

One of such hypothesis is that the organizational effect of the exposure to androgens in uterus might not result in the higher adult testosterone level but rather in greater hormonal reactivity to a situation perceived as a challenge, i.e. a fight or a physical effort^31^. However, there were only four studies in which the relationship between the 2D:4D and the testosterone change in response to a challenging (i.e. physical exertion, or aggressive context) situation was measured^20,32–34^, and they have led to equivocal results. In Kilduff *et al*.^33^ research, 25 professional rugby players performed a physical activity, resulting in a raise of the testosterone level. Results showed that there was no association between pre-exercise testosterone level and the 2D:4D, but the testosterone change was significantly correlated with the right (but not the left) digit ratio, meaning the lower the right 2D:4D, the higher the testosterone change under a physical effort. Kilduff *et al*.^34^ conducted also a similar research, but using psychological stimuli. 45 males watched an aggressive video or a blank screen in a cross-over design. Results showed that testosterone levels were higher after watching an aggressive video in comparison with a blank screen condition. This association was moderated by the left (but not the right) 2D:4D, meaning the lower the left 2D:4D, the greater change in testosterone. Another study^32^ investigated the relationship between the 2D:4D and a testosterone change after a combined physical exercise (repeated sprints), and psychological task (watching an aggressive movie) among 24 participants (12 males and 12 females). The right minus left (but neither the right, nor the left) 2D:4D was found to be negatively correlated with an increase in testosterone (but only in men). Interestingly, the difference between the right and the left 2D:4D (right minus left 2D:4D) seems to be sexually dimorphic, with lower values of right-left 2D:4D difference in men compared with women^35^, and this trait is also perceived to be a marker of prenatal testosterone level^36^. A most recent study^20^ provided an argument in favor of the hypothesis that the aforementioned relationship appears also among women. Crewther and Cook^20^ examined morning, basal testosterone, and testosterone changes under a physical exercise and psychological challenge among 35 physically active women. Authors indicated that training hours and phase in menstrual cycle moderated the association between testosterone and the right (the left was not measured) 2D:4D.

In the present study, we sought to extend the results of previous work on the relationship between the 2D:4D ratio and the testosterone change. Due to the fact that there are only four studies on this matter (among which one was carried only on female participants), conducted on relatively small samples, reporting conflicting results (the right 2D:4D vs the left 2D:4D vs the right minus left 2D:4D), we aimed to conduct an experiment on a large sample to investigate, whether digit ratios can be associated with a testosterone response to an acute exercise. Additionally, as previous studies on the physical exercise and the 2D:4D^20,32–34^ have not taken into consideration the possible influence of cortisol on testosterone activity (according to the assumptions of the dual-hormone hypothesis, testosterone may act differently in dependence of cortisol^37^; also prenatal androgen levels may influence the HPA axis, and the cortisol secretion in the adult life^38–41^, we investigated both the testosterone and the cortisol change. We also controlled other factors that may have influenced the sex hormonal levels: age^39,42^, BMI^43,44^, heart rate change^45,46^ (as an indicator of the intensity of a participant’s physical effort during the experiment), participants’ training experience (measured by the usual week’s physical activity)^47–49^. Based on previous studies^20,32–34^, we hypothesize that digit ratios are related to the testosterone spike.

## Results

### Descriptive statistics

We collected from participants the following information: age (mean = 21.29, *SD* = 1.81); height and weight (which was computed as BMI [weight (kg)/[height (m)]^2^], mean = 23.94, *SD* = 2.84); physical activity during a typical week (number of trainings per week*typical length of a training, mean = 5.42, *SD* = 4.45). We also measured: pre-exercise testosterone [pg/ml] (mean = 133.39, *SD* = 54.86); pre-exercise cortisol [ng/ml] (mean = 8.08, *SD* = 1.71); testosterone after the physical exertion [pg/ml] (mean = 145.85, SD = 57.92); cortisol after the physical exertion [ng/ml] (mean = 7.88, *SD* = 1.83); testosterone change [pg/ml] (mean = 12.45, *SD* = 45.52); cortisol change [ng/ml] (mean = −0.19, *SD* = 1.59); pre-exercise heart rate [bpm] (mean = 68.40, *SD* = 9.53); heart rate [bpm] after 5^th^ sprint (mean = 166.00, *SD* = 12.20); heart rate [bpm] change (mean = 97.49, *SD* = 13.68).

### Hormonal response to an acute exercise

There was a significant difference in the log testosterone level before and after the physical exertion (*t*(96) = −2.69, *p* < 0.01). Additionally, there was no difference in the log cortisol level before and after the physical exertion (*t*(96) = 1.38, *p* = 0.17). We found a positive correlation between the log testosterone change and the log cortisol change (*r* = 0.29, *p* < 0.01). In all subsequent analyses we used log transformed values of testosterone and cortisol. Due to the fact that there were seven outliers (above two standard deviations from the mean) in the testosterone change, we excluded outliers and conducted all analyses with remaining data (*N* = 90) (as suggested by, for instance, Pollet and van der Meij^50^). Nevertheless, obtained results were similar to the ones without the exclusions, thus, we decided not to remove them from the dataset.

### 2D:4D and the pre-exercise log testosterone level

Standard regression models were statistically significant for the right 2D:4D (Table 1 – Model 1), for the left 2D:4D (Table 1 – Model 2), and for the right minus left (Table 1 – Model 3). However, only the pre-exercise cortisol was a significant predictor of the pre-exercise testosterone (Table 1 - Model 1,2,3), whereas the 2D:4D, age, BMI, and physical activity during a typical week were not related with the pre-exercise testosterone. A summary of the regression results is presented in Table 1. Standard regression models for digit ratios and log testosterone without covariates are presented in supplementary material (Supplementary Table 1), under the link: https://figshare.com/s/9cdbafc8fd46f18b3e80. Standard regression models for digit ratios and log testosterone among participants who were less physically active, and more physically active are also presented in supplementary material (Supplementary Table 2, 3, accordingly). In all analyses, digit ratios were non-significant predictors of testosterone levels.

**Table 1:** A summary of the pre-exercise testosterone regression results. Significant results are bolded (N = 97).

	Model 1 Right 2D:4D			Model 2 Left 2D:4D			Model 3 Right-Left 2D:4D		
	Adj. *r*^2^ = 0.12, *F*(5,91) = 3.65, *p* < 0.01			Adj. *r*^2^ = 0.12, *F*(5, 91) = 3.58, *p* < 0.01			Adj. *r*^2^ = 0.12, *F*(5,91) = 3.57, *p* < 0.01		
	*β*	*t*	*p*	*β*	*t*	*p*	*β*	*t*	*p*
Basal cortisol	**0.39**	**3.98**	**<0.001**	**0.39**	**3.89**	**<0.001**	**0.38**	**3.84**	**<0.001**
2D:4D	−0.07	−0.70	0.49	−0.04	−0.40	0.69	−0.04	−0.36	0.72
Age	−0.09	−0.90	0.37	−0.09	−0.90	0.37	−0.09	−0.86	0.39
BMI*	−0.07	−0.72	0.48	−0.08	−0.78	0.44	−0.07	−0.68	0.50
Physical act.**	−0.04	−0.39	0.70	−0.04	−0.36	0.72	−0.04	−0.37	0.71

^*^BMI – Body Mass Index; **Physical act. – physical activity during a typical week (number of trainings per week*typical length of a training).

### 2D:4D and the log testosterone change in response to an acute exercise

Standard regression models were statistically non-significant for the right 2D:4D (Table 2 – Model 1), for the left 2D:4D (Table 2 – Model 2), and for the right minus left 2D:4D (Table 2 – Model 3). Only the cortisol change was a significant predictor of the testosterone change, whereas the 2D:4D, age, BMI, and physical activity during a typical week were not related with testosterone change in response to a physical exertion. A summary of the results of the three regression models is presented in Table 2. Standard regression models for digit ratios and log testosterone without covariates are presented in supplementary materials (Supplementary Table 4). Standard regression models for digit ratios and log testosterone among participants who were less physically active, and more physically active are also presented in supplementary material (Supplementary Table 5, 6, accordingly). In all analyses, digit ratios were non-significant predictors of testosterone levels).

**Table 2:** A summary of the testosterone change regression results. Significant results are bolded (N = 97).

	Model 1 Right 2D:4D			Model 2 Left 2D:4D			Model 3 Right-Left 2D:4D		
	Adj. *r*^2^ = 0.05, *F*(6,90) = 1.79, *p* = 0.11			Adj. *r*^2^ = 0.05, *F*(6,90) = 1.79, *p* = 0.11			Adj. *r*^2^ = 0.05, *F*(6,90) = 1.78, *p* = 0.11		
	*β*	*t*	*p*	*β*	*t*	*p*	*β*	*t*	*p*
Cortisol change	**0.28**	**2.73**	**<0.01**	**0.28**	**2.68**	**<0.01**	**0.29**	**2.72**	**<0.01**
Digit ratios	−0.02	−0.23	0.82	−0.02	−0.22	0.83	0.00	0.004	0.99
HR change*	0.04	0.38	0.71	0.04	0.40	0.69	0.04	0.39	0.70
Age	−0.03	−0.30	0.76	−0.03	−0.31	0.76	−0.03	−0.30	0.77
BMI**	−0.12	−1.17	0.25	−0.13	−1.19	0.24	−0.12	−1.16	0.25
Physical Act.***	0.01	0.12	0.90	0.01	0.13	0.90	0.01	0.14	0.89

^*^HR change – Heart Rate change; **BMI – Body Mass Index; *** Physical act. – physical activity during a typical week (number of trainings per week *typical length of a training).

## Discussion

The results of our study did not confirm the hypothesized relationship between the 2D:4D and a change in a testosterone level in response to an acute exercise. We found that the digit ratios (the right 2D:4D, the left 2D:4D, and the right minus left 2D:4D) were neither predictors of pre-exercise testosterone, nor the testosterone change after the physical exertion.

Our findings contradict previous studies^20,33^, which provided evidence that the 2D:4D is a predictor of the change in the testosterone level after a physical effort. Some studies have also indicated such relationship after an aggressive stimuli^34^, and a physical effort combined with an aggressive stimuli^32^, but, as our study did not include an aggressive context, it is not an exact replication of all previous research – we focused solely on the physical exertion. However, this should not affect the investigated effects because in previous studies (e.g.,^51–53^) it was demonstrated that intense physical effort alone leads to hormonal changes, including a significant increase in the testosterone level.

One of the strengths of our study is the sample size (*N* = 97). The number of participants in our research is larger than the number of participants in all three previous studies (on the link between the 2D:4D and a testosterone change in response to a challenging situation (i.e. acute exercise or aggressive context) combined (^20^ *N* = 35;^32^ *N* = 24;^33^ *N* = 25). This may suggest that the 2D:4D is not as strong predictor of the impact of organizational effect of an intrauterine testosterone level on a testosterone response to a challenging situation in adulthood, as have been previously thought.

Our results provide evidence for the lack of the relationship between the testosterone level and the 2D:4D in adult men, what is in line with previous studies^17,24–26,33^. This suggests that the 2D:4D, even if related with prenatal testosterone level, should not be used as a proxy of the adult testosterone level. The adult testosterone level is related with many aspects of a man’s lifestyle, such as, for instance, smoking^54^, body weight^55^, diet^56^, that may act on various stages of ontogenesis and impact the relationship between the 2D:4D and the adult testosterone level.

We also explored the possible effect of cortisol activity on the relationship between the 2D:4D and a testosterone response to a physical exertion. Both hormones, testosterone and cortisol, are expected to increase in response to a physical activity^57–59^ (but see also^60^), and cortisol has been shown to influence a testosterone secretion and activity^39–41^, thus, cortisol may impact the relationship between the 2D:4D and the adult testosterone level or testosterone response to a physical exertion. We found a positive relationship between a testosterone and cortisol change in response to an acute exercise, what was also shown in previous studies^57–59^, but we found no link between cortisol, testosterone, a testosterone change, and the 2D:4D. It may be that cortisol increases only in certain situations, perceived as stressful^61^, while our study, although physically demanding, did not elicit a psychologically stressful response.

One of the general limitations of studies on the digit ratios is the high number of degrees of freedom within the 2D:4D (the right, left, and right minus left 2D:4D). Running many analyses with different predictors increases the chances of finding allegedly significant results^62^. Bearing in mind that this may be a strength (allowing us to compare our results with previous studies), but also a caveat (of increasing chances of finding significant results), we decided to test all three 2D:4D. Our study seems to overcome this limitation, as the results show a similar pattern for all three digit ratios (i.e. none of the 2D:4D was a significant predictor of the androgen hormone levels).

Considering the fact that not only our, but also other studies do not support the hypothesis that the digit ratios are related to testosterone levels^28^, or even prenatal hormones^8,63,64^ (but see also^65^), it may seem surprising that the abundant body of literature provides evidence for the links between the 2D:4D and many adult characteristics, such as aggressiveness^66^, personality traits^30^, or substance and computer use^29^. What may seem even more confusing, is that some other, recent studies have reported the 2D:4D to be a non-significant predictor of other traits and behaviors, for instance: risk or pro-social behaviors^67,68^, risk taking^69^, grip strength^70^. One of the possible explanations of such discrepancies may be taking into account a size of an individual. It has been suggested that the digit ratios are lower among bigger individuals, and higher among smaller individuals^71^, as lengths of fingers do not grow proportionally – while a whole body is growing, a fourth digit elongates slightly faster than a second digit^72^. Thus, some of the positive findings on testosterone and the 2D:4D links may be merely due to effects of allometry, and not the effects of hormones on the digit ratio differences^73^. We conclude that studies on the 2D:4D and their correlates should always control for the size of participants’ bodies.

In conclusion, the present study sought to investigate the relationship between the digit ratios and both pre-exercise testosterone, and a testosterone change in response to an acute exercise. Our findings did not provide evidence for such links, what is contradictive to previous studies^20,32,33^. Interestingly, research involving digit ratios are still thriving, by showing significant relationships with other characteristics^30,66^, yet, more and more scholars express their concerns about the validity of the 2D:4D as the potential markers of the organizational effect of the exposure to androgens in uterus^73,74^, we believe that there is yet much to discover regarding the origins and reliable correlates of the 2D:4D. To bring conclusive arguments, future studies should be conducted on large and various populations (e.g., not only physically active persons or sports professionals), controlling for a body size, and, ideally, pre-registered.

## Materials and method

### Participants

Healthy, young, and physically active men were recruited into the study. Participants were recruited via leaflets, social media, and direct invitations by researchers during physical activity courses. Only participants who meet the inclusion criteria (i.e. no current or recent infections; no chronic disease, e.g., allergies, asthma, diabetes; not taking hormonal treatment or hormonal supplements; not smoking and drinking alcohol 24 hours prior to the testing; no finger injuries in the past that may influence the digit ratios; no injuries in the mouth cavity that may result in saliva sample contamination with blood, e.g., braces, recent dental treatments, erupting eight teeth) participated in the study: 97 men, aged 19–25 (mean = 21.29; *SD* = 1.54). Each participant provided informed written consent to participate in the study. The study was in accordance with guidelines of Declaration of Helsinki. Moreover, ethical approval for conducting the study has been granted by the Institutional Ethics Committee at the Institute of Psychology at the University of Wrocław.

### General procedure and questionnaires

**Figure 1:**
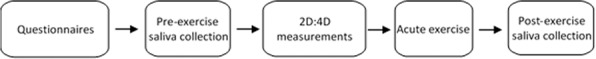
The study protocol.

Our study protocol is presented in the Fig. 1. The experiment was conducted between 7 AM and 11 AM in order to minimize the diurnal hormones fluctuations (^75^. In order to reduce any bias in salivary testosterone, participants were asked to refrain from physical activity for 24 h, eating and brushing their teeth 2 h prior to the study^20^.

Before the experiment, participants answered the questionnaire, reporting their age, how often they exercise and type of that activity, average duration and intensity of their trainings, their body height, and weight (which was also measured right before the physical exertion). In all subsequent analysis we used participants’ BMI, calculated based on self-reported weight and height (weight (kg) / [height (m)]^2^).

Participants’ heart rate was also measured (after filling the first questionnaire, which served as a basal heart rate, and after the 5^th^ sprint on the bike, representing the heart rate peak), using sport-tester Polar S810 (Polar Electro, Finland). The heart rate change (subtraction of the basal heart rate from the heart rate peak) was included in the analysis as the measure of the participants’ physical effort^45,46^.

### Digit ratio measurement

The lengths of the 2^nd^ and 4^th^ digits in both hands were measured directly with a digital calipers (Verke V86000), to a resolution of 0.01 mm. Measurements were made from a mid-point on the crease proximal to the palm to the end of the finger (as in^5^). Two measurements for each hand were performed. The intra-class correlation coefficients (*ICC*) for the first and second measurements of digit ratios for the right hand were *ICC* = 0.89, and for the left hand *ICC* = 0.89. We also conducted ANOVA tests for repeated measures, and the analysis showed that the measurement error of the 2D:4D was much smaller than between-individual differences (for the right hand: *F* = 17.255, *p* < 0.001; for the left hand: *F* = 17,732, *p* < 0.001). Therefore, in all subsequent analyses we used means of the 2D:4D from the first and second measurements (mean for the right 2D:4D: 0.978 (*SD* = 0.030); for the left 2D:4D: 0.983 (*SD* = 0.032); and for the right minus left 2D:4D: −0.005 (*SD* = 0.024)).

### Salivary hormones assessment

Testosterone and cortisol concentrations were measured in saliva^75^. At each saliva collection point, participants provided two 1 ml saliva samples by passive drool into two 2 ml container (two samples per one collection, in total four samples), which was then stored at – 17 °C for maximum a week, and then, the samples were transported to the lab and stored at −80 °C until the further analysis. Prior the first saliva collection participants drank 100 ml of water in order to increase saliva flow. The first saliva collection was approximately 10 minutes before a physical exertion. The second sample collection started 12 minutes after the end of the physical exertion (as this time window is typically associated with the expected peak in the testosterone change^76,77^, and after drinking another 100 ml of water (see Fig. 1). Participants were encouraged to finish the task of filling the saliva vials as fast as possible.

Hormonal analyses were performed in duplicate, within two months of the saliva samples collection using competitive enzyme linked immunosorbent assay (ELISA method). Before the analyses, samples were thawed and centrifuged for 10 minutes at 10 000 RPM. Clear supernatant was used to quantitative determination of free testosterone and free cortisol by commercial ELISA kit (DES6622 and DES6611, DEMEDITEC). Intra- and inter-coefficient of variations were respectively: <9.7%; <9.9% for fT (free testosterone) and <6.8%; <9.4% for C (cortisol) with assay sensitivity 2.2 pg/ml for fT and 0.014 ng/ml for C. Assay procedure and calculation of results were carried out in accordance to user’s manual. The concentrations of hormones in assayed samples were expressed in pg/ml for fT and ng/ml for C.

### Acute exercise

**Figure 2:**
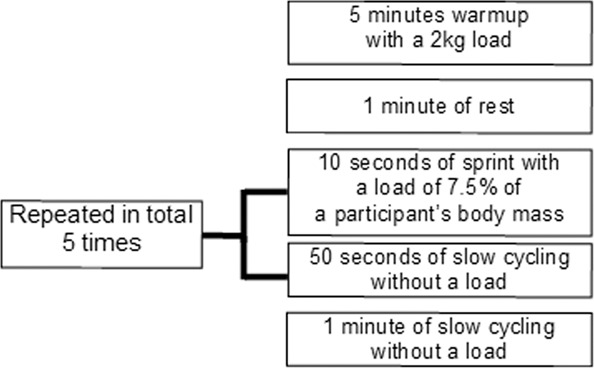
Acute exercise protocol.

The physical exertion consisted of a repeated sprint activity on a stationary bike (Monark 824E, Sweden), which followed the same scientific criteria as tools used in previous studies^32,78–80^. Prior to the start of the test, each participant was weighed to ensure a proper adjustment of the training load. Figure 2 displays the cycling protocol.

### Statistical analyses

Analyses were performed in Statistica (12.0) and Jamovi (version 1.0)^81^.

Log transformed testosterone and cortisol preceding and after an acute exercise were compared with a paired-samples t-test.

Standard multiple regression analyses were conducted to assess the relationship between both pre-exercise testosterone (Table 1), and a change in testosterone (Table 2), and the 2D:4D, cortisol, age, BMI, physical activity during a typical week (number of trainings per week*typical length of a training), heart rate change (heart rate after 5th sprint minus basal heart rate; this variable was used only in a testosterone change model). Three models were computed separately for the right 2D:4D, the left 2D:4D, and the right minus left 2D:4D (as reported in previous studies, e.g.,^32^). Standard multiple regression analyses were conducted to assess the relationship between: pre-exercise testosterone (Supplementary Table 1), and a change in testosterone (Supplementary Table 4), and the 2D:4D without other covariates. As the amount of physical activity influences testosterone levels^47–49^, a median split was performed to distinguish less and more physically active persons. Standard multiple regression analyses were conducted to assess the relationship between: pre-exercise testosterone and digit ratios among less physically active participants (Supplementary Table 2), and more active participants (Supplementary Table 3). Similarly, standard multiple regressions analyses were performed to asses the relationship between a testosterone change and digit ratios among less physically active participants (Supplementary Table 5), and more physically active participants (Supplementary Table 6). All additional analyses are reported in supplementary material, which can be found under the link: https://figshare.com/s/9cdbafc8fd46f18b3e80.

## Supplementary information

Supplementary information.

Supplementary information2.
