# Supplementary material for: No relationship between the digit ratios (2D:4D) and salivary testosterone change: Study on men under an acute exercise
Source: Sci Rep. 2020 Jun 22;10:10068. doi: 10.1038/s41598-020-66915-9 (PMC7308391; doi:10.1038/s41598-020-66915-9)
Supplement: Supplementary file 2 — Supplementary information2. [file 41598_2020_66915_MOESM2_ESM.docx]

**No relationship between the digit ratios (2D:4D) and salivary testosterone change: Study on men under an acute exercise**

Marta Kowal^1^, Piotr Sorokowski^1*^, Agnieszka Żelaźniewicz^2^, Judyta Nowak^2^, Sylwester Orzechowski^1^, Grzegorz Żurek^3^, Alina Żurek^1^, Anna Juszkiewicz^1^, Lidia Wojtycka^1^, Wiktoria Sieniuć^1^, Małgorzata Poniatowska^1^, Karolina Tarnowska^1^, Kaja Kowalska^1^, Katarzyna Drabik^1^, Patrycja Łukaszek^1^, Krzysztof Krawczyk^4^, Tadeusz Stefaniak^3^, Natalia Danek^3^.

^1^Institute of Psychology, University of Wrocław, Wrocław, Poland.
^2^Department of Human Biology, University of Wrocław, Wrocław, Poland.
^3^Wroclaw Sports University, Wroclaw, Poland.
^4^Maria Curie-Skłodowska University, Lublin, Poland.

***Correspondence:**Piotr Sorokowski
sorokowskipiotr@yahoo.co.uk

**Supplementary material**

Table 1. A summary of the pre-exercise testosterone regression results (N = 97).

|  | Model 1  Right 2D:4D | | | Model 2  Left 2D:4D | | | Model 3  Right-Left 2D:4D | | |
| --- | --- | --- | --- | --- | --- | --- | --- | --- | --- |
|  | Adj. *r^2^* = -.01z, *F*(1,95) = .00, *p* = .99 | | | Adj. *r^2^* = -.01, *F*(1, 95) = .45, *p* = .50 | | | Adj. *r^2^* = -.00, *F*(1,95) = .84, *p* = .36 | | |
|  | β | *t* | *p* | β | *t* | *p* | β | *t* | *p* |
| 2D:4D | -.00 | -.02 | .99 | .07 | .67 | .50 | -.09 | -.92 | .36 |

Table 2. A summary of the pre-exercise testosterone regression results for less physically active participants (below median; N = 48).

|  | Model 1  Right 2D:4D | | | Model 2  Left 2D:4D | | | Model 3  Right-Left 2D:4D | | |
| --- | --- | --- | --- | --- | --- | --- | --- | --- | --- |
|  | Adj. *r^2^* = .12, *F*(2,45) = 4.29, *p* < .05 | | | Adj. *r^2^* = .12, *F*(2,45) = 4.24, *p* < .05 | | | Adj. *r^2^* = .13, *F*(2,45) = 4.36, *p* < .05 | | |
|  | β | *t* | *p* | β | *t* | *p* | β | *t* | *p* |
| Basal cortisol | .39 | 2.84 | < .01 | .39 | 2.87 | < .01 | .37 | 2.52 | < .05 |
| 2D:4D | -.04 | -.31 | .76 | .01 | .03 | .97 | -.07 | -.46 | .65 |

Table 3. A summary of the pre-exercise testosterone regression results for more physically active participants (above median; N = 35).

|  | Model 1  Right 2D:4D | | | Model 2  Left 2D:4D | | | Model 3  Right-Left 2D:4D | | |
| --- | --- | --- | --- | --- | --- | --- | --- | --- | --- |
|  | Adj. *r^2^* = .11, *F*(2,32) = 2.98, *p* = .07 | | | Adj. *r^2^* = .08, *F*(2,32) = 2.55, *p* =.09 | | | Adj. *r^2^* = .07, *F*(2,32) = 2.32., *p* =.11 | | |
|  | β | *t* | *p* | β | *t* | *p* | β | *t* | *p* |
| Basal cortisol | .45 | 2.44 | < .05 | .38 | 2.25 | < .05 | .37 | 2.14 | <.05 |
| 2D:4D | -.21 | -1.14 | .26 | -.12 | -.73 | .47 | -.07 | -.38 | .71 |

 Table 4*. A summary of the testosterone change regression results (N = 97).*

|  | Model 1  Right 2D:4D | | | Model 2  Left 2D:4D | | | Model 3  Right-Left 2D:4D | | |
| --- | --- | --- | --- | --- | --- | --- | --- | --- | --- |
|  | Adj. *r^2^* = -.01, *F*(1,95) = .32, *p* = .58 | | | Adj. *r^2^* = -.01, *F*(1,95) = .29, *p* = .59 | | | Adj. *r^2^* = -.01, *F*(1,95) = .00, *p* = .99 | | |
|  | β | *t* | *p* | β | *t* | *p* | β | *t* | *p* |
| 2D:4D | -.06 | -.56 | .58 | -.06 | -.54 | .59 | .00 | .01 | .99 |

Table 5. A summary of the testosterone change regression results for less physically active participants (below median; N = 48)

|  | Model 1  Right 2D:4D | | | Model 2  Left 2D:4D | | | Model 3  Right-Left 2D:4D | | |
| --- | --- | --- | --- | --- | --- | --- | --- | --- | --- |
|  | Adj. *r^2^* = .06, *F*(2,45) = 2.46, *p* = .09 | | | Adj. *r^2^* = .06, *F*(2,45) = 2.59, *p* = .09 | | | Adj. *r^2^* = .06, *F*(2,45) = 2.57, *p* = .09 | | |
|  | β | *t* | *p* | β | *t* | *p* | β | *t* | *p* |
| Cortisol change | .31 | 2.22 | <.05 | .30 | 2.13 | < .05 | .29 | 2.06 | < .05 |
| Digit ratios | -.03 | -.17 | .86 | -.08 | -.53 | .59 | .07 | .48 | .63 |

Table 6. A summary of the testosterone change regression results for more physically active participants (above median; N = 35).

|  | Model 1  Right 2D:4D | | | Model 2  Left 2D:4D | | | Model 3  Right-Left 2D:4D | | |
| --- | --- | --- | --- | --- | --- | --- | --- | --- | --- |
|  | Adj. *r^2^* = .03, *F*(2,32) = 1.49, *p* = .24 | | | Adj. *r^2^* = .02, *F*(2,32) = 1.35, *p* = .27 | | | Adj. *r^2^* = .04, *F*(2,32) = 1.73, *p* = .19 | | |
|  | β | *t* | *p* | β | *t* | *p* | β | *t* | *p* |
| Cortisol change | .25 | 1.41 | .17 | .28 | 1.64 | .11 | .25 | 1.46 | .16 |
| Digit ratios | -.09 | -.54 | .59 | .02 | .13 | .90 | -.15 | -.85 | .41 |
